# Effects of Different Components of PM_2.5_ on the Expression Levels of NF-κB Family Gene mRNA and Inflammatory Molecules in Human Macrophage

**DOI:** 10.3390/ijerph16081408

**Published:** 2019-04-19

**Authors:** Jian Zhu, Yaming Zhao, Yizhen Gao, Chunyan Li, Liting Zhou, Wen Qi, Yuezhu Zhang, Lin Ye

**Affiliations:** 1 Department of Occupational and Environmental Health, School of Public Health, Jilin University, Changchun 130000, China; zhujian@jlu.edu.cn (J.Z.); zhaoymtg@163.com (Y.Z.); gyz17843129095@163.com (Y.G.); zhoulttg@163.com (L.Z.); qiwen16@mails.jlu.edu.cn (W.Q.); s418824079@126.com (Y.Z.); 2 Clinical Teaching and Research Laboratory, Medical School, Xilingol Vocational College, Inner Mongolia 026000, China; 15033359433@163.com

**Keywords:** different components of PM_2.5_, cytotoxicity, NF-*κ*B, inflammatory molecules

## Abstract

*Background:* Studies have found that exposure to fine particulate matter with sizes below 2.5 µm (PM_2.5_) might cause inflammation response via the NF-*κ*B pathway. To date, only a few studies have focused on the toxicity of different components of PM_2.5_. We aimed to explore the effects of PM_2.5_ with different components on the expression levels of NF-*κ*B family gene mRNA and inflammatory molecules in human macrophages. *Methods:* Human monocytic cell line THP-1-derived macrophages were exposed to water-soluble (W-PM_2.5_), fat-soluble (F-PM_2.5_), and insoluble (I-PM_2.5_) PM_2.5_. The cell survival rate was measured by 3-(4,5-dimethylthiazol-2-yl)-2,5-diphenyltetrazolium bromide (MTT) assay. The levels of inflammatory molecules were determined by enzyme-linked immunosorbent assay (ELISA), and the relative mRNA levels of the NF-*κ*B family gene were determined by real time PCR. *Results:* PM_2.5_ could decrease the cell viability. After exposure to W-PM_2.5_, the levels of interleukins (IL)-1β and IL-12 p70 significantly increased. After exposure to F-PM_2.5_, the levels of IL-12 p70 significantly increased. The levels of IL-12 p70 and TNF-α after exposure to I-PM_2.5_ were significantly higher than that in W- and F-PM_2.5_ treatment groups. The levels of IL-8, C reactive protein (CRP), and cyclooxygenase (COX)-2 increased only after exposure to I-PM_2.5_. F-PM_2.5_ increased the mRNA levels of NF-*κ*B genes, especially *NF*-*κB_1_* and *RelA*. *Conclusions:* PM_2.5_ can decrease the cell survival rate and up-regulate the expression of NF-*κ*B family gene mRNA and inflammatory molecules. The main toxic components of PM_2.5_ related to inflammatory response in macrophages were the I-PM_2.5_.

## 1. Introduction

Epidemiological and experimental studies have proven that exposure to atmospheric particulate matter (PM), especially exposure to fine particulate matter with sizes below 2.5 µm (PM_2.5_), is closely associated with population mortality [1,2,3]. PM is considered to have contributed to the global burden of respiratory system diseases and cardiovascular diseases [4]. This is because PM_2.5_ can be inhaled deeply into the lung alveoli, and some of them can even enter the bloodstream [5].

PM_2.5_ can induce many pro-inflammatory responses in vivo, including increasing the production of reactive oxygen species (ROS), altering the transcription of inflammation-related genes and the polarization of macrophages, and overproducing pro-inflammatory molecules [6,7,8]. PM_2.5_ can cause inflammatory responses and oxidative stress in human bronchial epithelial cells (16HBE) and human lung epithelial type 2 cells (A549) in vitro and in rat lungs in vivo [9,10,11]. Zhao et al. found that PM_2.5_ could induce increasing levels of interleukin (IL)-6, IL-1β and tumor necrosis factor-α (TNF-α) in macrophages [12]. PM_2.5_ also increases the levels of IL-1β, IL-6, IL-8, and cyclooxygenase (COX)-2 in a dose-dependent manner [13]. Exposure to PM_2.5_ can induce the release of proinflammatory molecules, such as IL-8 in bronchial epithelial cells [14]. In addition, the mRNA expression levels of IL-6, IL-1β, and IL-5 in liver of mice were also up-regulated after being exposed to PM_2.5_ [15]. The PM_2.5_-induced pro-inflammatory responses were considered the main reason for respiratory damage [16]. Exposure to PM_2.5_ can activate the nuclear factor-*κ*B (NF-*κ*B) pathway [17,18,19], and up-regulate the expression levels of NF-*κ*B_1_ and RelA and inflammatory molecules such as IL-1β amd IL-6 in human bronchial epithelial cells [13,20].

NF-*κ*B is known to be a critical signal pathway regulating immunity and inflammation [21]. The NF-*κ*B family includes NF-*κ*B_1_, NF-*κ*B_2_, RelA, RelB, and c-Rel. On one hand, NF-*κ*B can regulate gene transcriptions of pro-inflammatory molecules and chemokines, such as IL-1β, IL-6, and TNF-α, and inflammatory enzymes, such as COX-2 and inducible nitric oxide synthase (iNOS) [22]. On the other hand, inflammatory action can promote the activation of the NF-*κ*B signal pathway. Certain NF-*κ*B-dependent pro-inflammatory molecules, such as TNF-α, IL-1β and IL-6, can promote acute or chronic inflammation via the NF-*κ*B signaling pathway [23]. When PM_2.5_ reaches the alveoli through the trachea, the alveolar macrophages can phagocytose the particles and secrete inflammatory molecules. The NF-*κ*B signaling pathway is activated and participates in the inflammatory response.

The sources of PM_2.5_ are very wide-ranging, and the components are complex (sulfate, nitrate, organic chemicals, metals, crystal elements, etc.) [24]. Current studies mainly focus on the toxic effects of total PM_2.5_, and only a few studies have explored the effects of different components of PM_2.5_ [25,26]. The mechanism of inflammation caused by different components of PM_2.5_ remains to be studied.

Therefore, the present study explored the effects of different components of PM_2.5_ on the levels of NF-*κ*B family gene mRNA expression and inflammatory molecules in human monocytic cell (THP-1)-derived macrophages. This study contributes to fully understanding the toxicity of PM_2.5_, and provides a basis for the prevention and treatment of respiratory diseases caused by different types of PM_2.5_.

## 2. Materials and Methods

### 2.1. PM_2.5_ Sampling and Constituent Preparation

#### 2.1.1. PM_2.5_ Sampling

Ambient PM_2.5_ was collected from one of Changchun City’s PM_2.5_ monitoring points between March and June 2015, using an air sampler with a flow rate of 100 L/min. The microfiber filters were replaced every 24 h. The standardized pressure, humidity, and sample volume were recorded. After sampling, the microfiber filters were dried for 24 h at a stable temperature and weighed. The filters were then marked and stored in sealed bags at −20 °C.

#### 2.1.2. PM_2.5_ Component Extraction and Solution Preparation

The microfiber filters containing PM_2.5_ were irradiated by ultraviolet radiation for 30 min and cut into pieces with a diameter of 1 cm. The pieces were immersed in deionized water, vibrated by ultrasonic concussion for 30 min, and filtered to form the filtrates which were lyophilized to powder. The PM_2.5_ collected from all microfiber filters in the three months was mixed for subsequent sample preparation. The blank microfiber filters were processed in the same way. The blank microfiber filters were diluted with sterile phosphate-buffered saline (PBS, GIBCO, Grand Island, NY, USA) as control. A certain amount of PM_2.5_ powder was diluted by sterile PBS to form a suspension with a concentration of 1000 μg/mL. The PM_2.5_ suspension was centrifuged at 13,000 rpm for 30 min, and the supernatant was collected as the water-soluble PM_2.5_ (W-PM_2.5_). After centrifugation, the precipitates were freeze-dried and weighed. One hundred percent (100%) dimethylsulfoxide (DMSO) was used to prepare the fat-soluble PM_2.5_ (F-PM_2.5_), with a concentration of 20 mg/mL diluted by the culture medium. Ultrasound was used for 60 min to fully elute organic components attached to PM_2.5_ particles. The precipitate was vacuumized and dried again, dissolved in sterilized PBS solution, and vibrated by ultrasound for 30 min to prepare the insoluble PM_2.5_ (I-PM_2.5_) with a concentration of 1000 μg/mL. Different components of PM_2.5_ were stored at 4 °C. The suspension was vibrated again before use and diluted to the desired concentration by RPMI 1640 supplemented with fetal bovine serum (FBS).

### 2.2. Cell Culture and Exposure to PM_2.5_

The human monocytic cell line THP-1 was purchased from the Shanghai Chinese Academy of Science cell bank. The THP-1 cells were subcultured using RPMI 1640 complete medium (GIBCO, Grand Island, NY, USA) containing 10% FBS (BI, Kibbutz Beit Haemek, Israel) at 37 °C and 5% CO_2_. The growth of cells was viewed under an inverted microscope. When the cells were in good condition and the concentration of the cells was adjusted to 1.0 × 10^6^/mL, phorbol myristate acetate (PMA, Sigma, St. Louis., MO, USA) was added to the cells to induce the cells for 72 h, and the final concentration was 100 ng/mL. The differentiation of cells was observed under a microscope. When the differentiation rate of cells was over 95%, the following experiment could be carried out. The differentiated cells were washed by sterilized PBS three times to remove PMA, and were randomly divided into control, water-soluble, fat-soluble, and insoluble. The cells of each condition were continuously treated with the corresponding PM_2.5_ components and concentrations (Table 1). In order to control the effect of DMSO on cell growth, the final concentration of DMSO was about 0.35% (<1%). The cell supernatant and cells were collected and marked at 12, 24, and 48 h after treatment, and were stored at −80 °C for the detection of inflammatory molecules and the extraction of mRNA.

### 2.3. MTT for the Cell Survival Rate

The cell survival rate was measured by 3-(4,5-dimethylthiazol-2-yl)-2,5-diphenyltetrazolium bromide (MTT) assay to evaluate the effects of W-PM_2.5_, F-PM_2.5_, and I-PM_2.5_. The cells were seeded in 96 well plates and then treated with different doses of various components of PM_2.5_ (Table 1) for 12, 24, and 48 h. After exposure, 20 μL of MTT (1 mg/mL in PBS) was added to each well and incubated continuously for 2 h at 37 °C. The cells were then treated with 100 μL of DMSO. The absorbance was measured at 570 nm using a microplate reader (Thermo MK3, Winosky, VT, USA). Data were expressed as a percentage of the value obtained for the solvent control (0.1% DMSO), which was set to 100%.

### 2.4. ELISA for Inflammatory Molecules and Cytokines in the Supernatant

After exposure to PM_2.5_ for 12 and 24 h, the supernatant was collected to detect the measurement of IL-1β, IL-6, IL-8, IL-12 p70, TNF-α, CRP, IL-2, and COX-2. The measurement of inflammatory molecules and cytokines in the supernatant was performed using ELISA kits (Shanghai Hengyuan Biological Technology Co., Ltd., Shanghai, China) according to the manufacturer’s instructions.

### 2.5. Real-Time Quantitative PCR for the Expression Levels of NF-κB Family Genes

Macrophages were treated with PM_2.5_ at different doses and types of PM_2.5_ for 24 h. After cell lysis, total RNA isolation was performed by Trizol (Ambion, Shanghai, China) extraction. The mRNA, used as the template, was reverse-transcribed into cDNA using primers (TaKaRa, Dalian, China) and reverse transcriptase to perform PCR amplification. Real-time PCR was conducted in the Step One Plus TM apparatus. The PCR parameters were set as Segment 1: 95 °C, 30 s, 1 cycle; Segment 2, 95 °C, 5 s, 60 °C, 30 s, 40 cycles; Segment 3, 95 °C, 15 s, 60 °C, 1 min, 95 °C, 15 s, 1 cycle. The relative mRNA levels were relatively quantified via 2^−ΔΔCT^. Table 2 is the sequence of primers that was used to amplify candidate genes and internal reference genes.

### 2.6. Statistical Analyses

All statistical analyses were performed with IBM SPSS software version 24.0 (IBM, Arnmonk, NY, USA). Data were expressed as mean ± SD for normal continuous variables. Two-way analysis of variance (ANOVA) was used to analyze the differences in mRNA expression levels and inflammatory cytokine levels among different PM_2.5_ components and concentration groups, and least significant difference or Tamhane test was used to compare the difference between two groups. The relationship between NF-*κ*B mRNA levels and inflammatory-molecule levels was analyzed by linear correlation analysis.

## 3. Results

### 3.1. Differentiation of Monocytes

**Figure 1:**
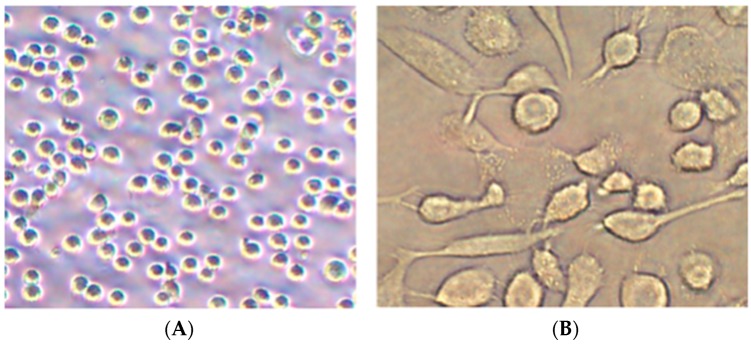
Morphology of THP-1 cells (**A**) before and (**B**) after differentiation.

The morphology of the cells was observed under microscope. The undifferentiated THP-1 cells were spherical and suspended (Figure 1A). After treatment with 100 ng/mL PMA for 72 h, mature macrophages differentiated from THP-1 cells adhered to the bottom of the flask and presented an irregular shape with pseudopod (Figure 1B).

### 3.2. Effects of PM_2.5_ on Cell Survival Rate

**Figure 2:**
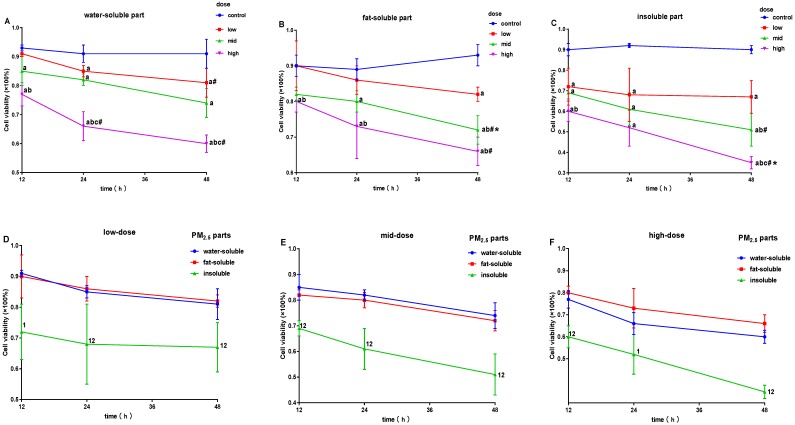
Effects of PM_2.5_ on the cell survival rate of macrophages. (**A**) Effects of W-PM_2.5_ on cell survival rates; (**B**) effects of F-PM_2.5_ on cell survival rates; (**C**) effects of I-PM_2.5_ on cell survival rates; (**D**) effects of low-dose PM_2.5_ on cell survival rates; (**E**) effects of mid-dose PM_2.5_ on cell survival rates; (**F**) effects of high-dose PM_2.5_ on cell survival rates. Data are presented as means ± SD of three independent experiments. ^a^ *p* < 0.05 vs. control group at the same time; ^b^ *p* < 0.05 vs. low-dose group at the same time; ^c^ *p* < 0.05 vs. mid-dose group at the same time; ^#^ *p* < 0.05 vs. 12 h group at the same dose; * *p* < 0.05 vs. 24 h group at the same dose; ^1^ *p* < 0.05 vs. W-PM_2.5_ at the same dose; ^2^ *p* < 0.05 vs. F-PM_2.5_ at the same dose.

Figure 2 shows that the survival rates of cells significantly decreased after exposure to W-PM_2.5_, compared to the control, except for the low dose for 12 h (*p* < 0.05, Figure 2A). After exposure to F-PM_2.5_ for 24 and 48 h, cell survival rates were significantly lower than that of the control (*p* < 0.05, Figure 2B). After exposure to I-PM_2.5_, survival rates of cells decreased significantly (*p* < 0.05, Figure 2C). In addition, the cell survival rate decreased earlier and more dramatically after exposure to I-PM_2.5_ than W-PM_2.5_ or F-PM_2.5_ (*p* < 0.05, Figure 2D–F).

### 3.3. Effects of PM_2.5_ on the Levels of IL-1β, IL-6, IL-8, IL-12 p70, TNF-α, CRP, IL-2, and COX-2

**Figure 3:**
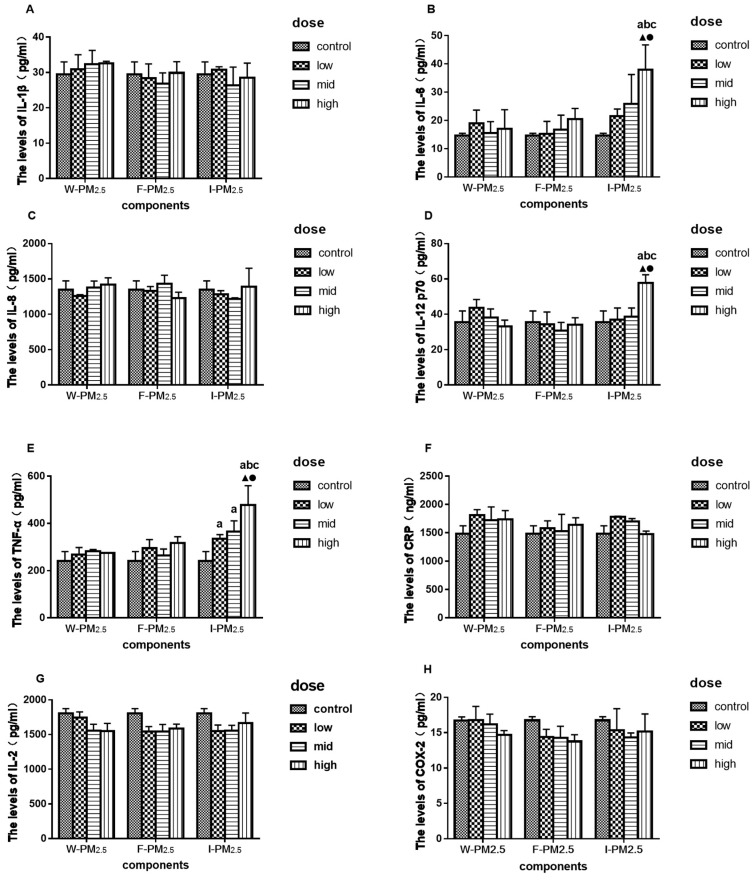
Effects of exposure to PM_2.5_ on the levels of IL-1β (**A**), IL-6 (**B**), IL-8 (**C**), IL-12 p70 (**D**), TNF-α (**E**), CRP (**F**), IL-2 (**G**), and COX-2 (**H**). Data are presented as means ± SD of three independent experiments. ^a^ *p* < 0.05 vs. control group; ^b^ *p* < 0.05 vs. low-dose group; ^c^ *p* < 0.05 vs. mid-dose group; ^▲^ *p* < 0.05 vs. W-PM_2.5_ at the same dose; ^●^ *p* < 0.05 vs. F-PM_2.5_ at the same dose.

Figure 3 shows the effects of exposure to PM_2.5_ for 12 h on the levels of inflammatory molecules. The levels of IL-6, IL-12 p70, and TNF-α increased significantly after exposure to a high dose of I-PM_2.5_ for 12 h (Figure 3B,D,E). After exposure to W- and F-PM_2.5_ for 12 h, levels of inflammatory molecules saw no significant changes.

**Figure 4:**
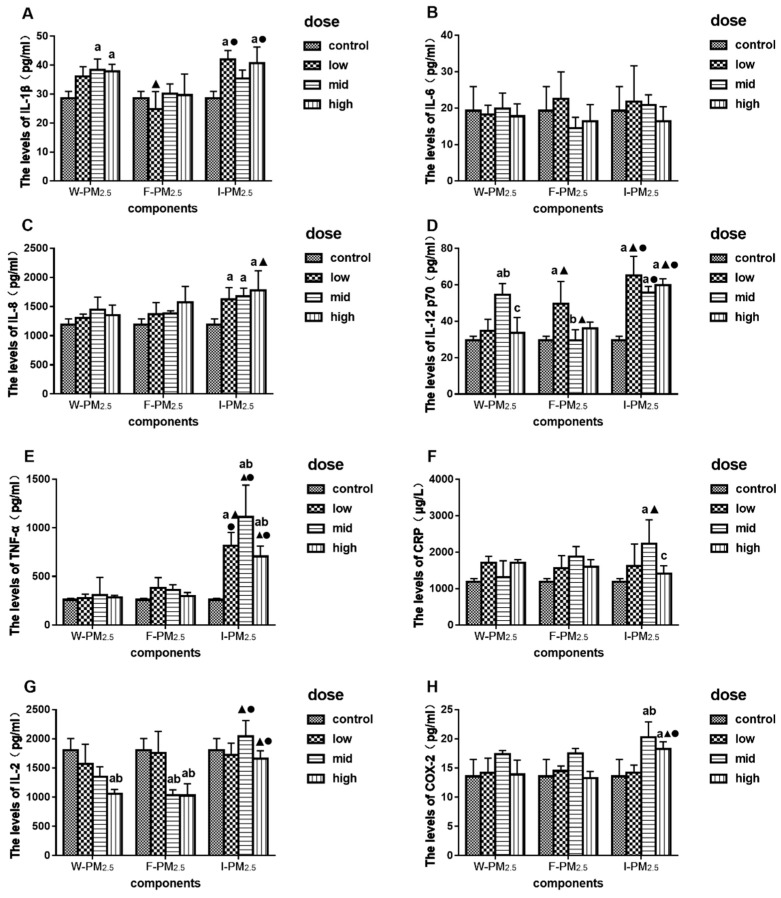
Effects of exposure to PM_2.5_ on the levels of IL-1β (**A**), IL-6 (**B**), IL-8 (**C**), IL-12 p70 (**D**), TNF-α (**E**), CRP (**F**), IL-2 (**G**), and COX-2 (**H**). Data are presented as means ± SD of three independent experiments. ^a^ *p* < 0.05 vs. control group; ^b^ *p* < 0.05 vs. low-dose group; ^c^ *p* < 0.05 vs. mid-dose group; ^▲^ *p* < 0.05 vs. W-PM_2.5_ at the same dose; ^●^ *p* < 0.05 vs. F-PM_2.5_ at the same dose.

Figure 4A–F shows the effects of exposure to PM_2.5_ for 24 h on the levels of inflammatory cytokines. After exposure to W-PM_2.5_, the expression levels of IL-1β significantly increased at mid and high doses, and the levels of IL-12 p70 were significantly higher than that of control at mid dose (Figure 4A,D, *p* < 0.05). After exposure to F-PM_2.5_, the expression levels of IL-12 p70 in the low-dose group were significantly higher than control (Figure 4D, *p* < 0.05). After exposure to I-PM_2.5_, the expression levels of IL-8, IL-12, and TNF-α significantly increased (Figure 4C–E, *p* < 0.05). The levels of IL-1β increased significantly after exposure to the low and high dose of I-PM_2.5_ (Figure 4A, *p* < 0.05). The levels of CRP significantly increased for the mid dose of I-PM_2.5_ (Figure 4F, *p* < 0.05).

In addition, the expression levels of IL-2 and COX-2 in the cell supernatant were detected and recorded. As shown in Figure 4G, the levels of IL-2 decreased significantly after exposure to the high dose of W- and F-PM_2.5_ (*p* < 0.05). The expression levels of COX-2 were significantly higher in the mid- and high-dose group after exposure to I-PM_2.5_ than that of control (Figure 4H, *p* < 0.05).

After exposure to I-PM_2.5_, the levels of IL-12 p70 and TNF-α were significantly higher than that in W- and F-PM_2.5_ treatment groups (Figure 4D, *p* < 0.05). The expression levels of IL-1β, after exposure to low and high doses of I-PM_2.5_, were significantly higher than that for the same dose of F-PM_2.5_ (Figure 4A, *p* < 0.05). Moreover, the levels of IL-8, CRP, and COX-2 increased only after exposure to I-PM_2.5_ (Figure 4C, F and H, *p* < 0.05).

### 3.4. Effects of PM_2.5_ on the Relative mRNA Expression Levels of NF-KB Family Gene

**Figure 5:**
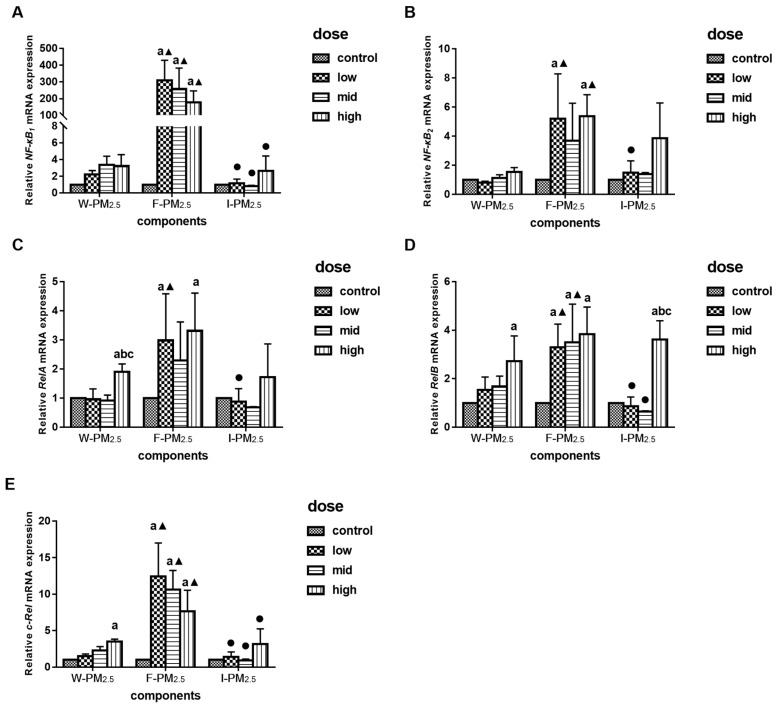
Effects of PM_2.5_ on the relative mRNA expression levels of *NF-κB_1_* (**A**), *NF-κB_2_* (**B**), *RelA* (**C**), *RelB* (**D**), and c-*Rel* (**E**). Data are presented as means ± SD of three independent experiments. ^a^ *p* < 0.05 vs. control group; ^b^ *p* < 0.05 vs. low-dose group; ^c^ *p* < 0.05 vs. mid-dose group; ^▲^ *p* < 0.05 vs. W-PM_2.5_ at the same dose; ^●^ *p* < 0.05 vs. F-PM_2.5_ at the same dose.

The relative mRNA expression levels of the NF-*κ*B signaling pathway related genes were measured after exposure to PM_2.5_ for 24 h. As shown in Figure 5C–E, when exposed to a high dose of W-PM_2.5_, the relative mRNA levels of *RelA*, *RelB*, and c-*Rel* were significantly higher than that of control (*p* < 0.05). Exposure to high dose of I-PM_2.5_ only resulted in the high expression of *RelB* (*p* < 0.05, Figure 5D). The low and high doses of F-PM_2.5_ increased the relative expressions of *NF-κB_2_* and *RelA*, whereas the relative levels of *NF*-*κB_1_*, *RelB*, and c-*Rel* increased at all concentrations (*p* < 0.05).

In conclusion, a high dose of W- and I-PM_2.5_ exposure increased the expression of several genes related to NF-*κ*B signaling pathways, but low-dose F-PM_2.5_ increased the mRNA levels of NF-*κ*B genes, especially *NF*-*κB_1_* and *RelA*.

### 3.5. The Connection between the Expression of NF-κB mRNA and Inflammatory Molecules

The correlation of the expression levels between NF-*κ*B signaling pathway mRNA and inflammatory molecules was analyzed to determine whether the exposure of different components of PM_2.5_ caused inflammation through the NF-*κ*B signaling pathway. After exposure to F-PM_2.5_, the expression levels of IL-8 were negatively correlated with c-*Rel*, whereas IL-12 p70 was positively correlated with *NF-κB_2_* and *c-Rel*. After treating the cells with I-PM_2.5_ for 24 h, the expression levels of IL-1 were positively correlated with *NF-κB_1_*, *NF-κB_2_*, and *RelB*, as well as IL-8 and *NF-κB_2_*. In addition, IL-6 was negatively correlated with the levels of NF-*κ*B genes (Table 3).

## 4. Discussion

PM_2.5_ are one of the important causes of respiratory diseases, cardiovascular diseases, and premature death [6,27,28,29]. Due to small particle sizes, PM_2.5_ can be easily inhaled and deposited into alveolar cells in the respiratory tract, causing or exacerbating lung diseases [30]. The compositions of PM_2.5_ are complex, and the chemical compositions are pathogenic [31]. Park et al. found that different components of PM_2.5_ had different effects on airway epithelial cells, and the combination components could cause respiratory diseases such as asthma [7]. The present study divided PM_2.5_ into W-PM_2.5_, F-PM_2.5_, and I-PM_2.5_ to investigate the macrophage toxicity of PM_2.5_ and its effect on the levels of the NF-*κ*B signaling pathway and inflammatory molecules, to analyze the mechanism of PM_2.5_-induced inflammation in respiratory diseases and to provide a new theoretical basis to prevent and treat the respiratory system diseases caused by atmospheric PM_2.5_.

Macrophages are the first barrier of the body’s immune system and the target cells for PM_2.5_ action. They can consume particles, secrete inflammatory molecules, and participate in various immune responses. In this study, THP-1-derived macrophages were selected as the research subjects according to the research objectives, and treatment was conducted with different PM_2.5_ components. Based on previous studies [32,33], we scaled the exposure doses. We chose higher doses of the total PM_2.5_ because we decomposed the total PM_2.5_.

Many studies have confirmed that PM_2.5_ has a toxic effect on cells [34,35,36]. The results of this study showed that all three components of PM_2.5_ led to a significant decrease in cell survival rates. The effect of I-PM_2.5_ was even more pronounced. The reason may be that I-PM_2.5_ leads to cell death through damage to the cell membrane and cell–particle interaction, whereas W-PM_2.5_ causes cell damage through the early response of oxidative stress. The former is more direct [26,37,38,39].

Water-soluble PM_2.5_ was mainly considered to comprise various inorganic salt ions and metal ions, including NO^3−^, SO_4_^2−^, NH^4+^ arsenic, Fe, Cu, zinc, and lead, in which soluble metal ions are regarded as the main cause of oxidative stress and lung damage [40,41]. Transition metals in PM promote the production and release of ROS, which cause or exacerbate inflammation [42]. The results of this experiment indicate that exposure to W-PM_2.5_ for 24 h could promote the expression levels of IL-1β and IL-12 p70. The former (IL-1β) is one of the most representative inflammatory molecules, and its high expression indicates that W-PM_2.5_ caused inflammation of macrophages, but it may be easily cleared by cells to eliminate inflammation. The latter (IL-12 p70) is an important immune regulatory cytokine and a member of a small family of heterodimeric cytokines [43]. The increase of IL-12 p70 expression could promote the development of inflammation [44].

Fat-soluble PM_2.5_ mainly comprises organic compounds that are harmful to the human body, such as polycyclic aromatic hydrocarbons and dioxins [16]. Fuentes-Mattei et al. have suggested that organic extract of PM_2.5_ possibly suppresses the role of pregnant X receptor (PXR) and CYP3A5 on human epithelial cells, which triggers an inflammatory response [45]. Metal not only exists in the aqueous extract of PM_2.5_, but can also be found in organic extracts. It is interesting to note that, according to reports, heavy metal in PM_2.5_ organic extracts becomes redox-active, and when there is a metal-chelating agent in airway epithelial cells, organic extracts release IL-6 and IL-8. Therefore, the combination of polycyclic aromatic hydrocarbons (PAHs) or polycyclic aromatic hydrocarbons and organic extraction metals such as Fe and Cu may help to promote inflammatory responses through ROS, thereby exacerbating respiratory disease. The results of this study show that the levels of IL-12 p70 increased significantly after exposure to F-PM_2.5_ for 24 h. Furthermore, the small changes of the inflammatory molecules caused by F-PM_2.5_ may mainly be due to its low dose.

Insoluble PM_2.5_ was the main component of total PM_2.5_, accounting for more than 50% of the total weight of PM_2.5_ [46]. The concentrations of PAHs and heavy metals were much higher in I-PM_2.5_ than in others, due to their tendency to partition the solid [47]. Additionally, larger total quantities of catalytically and biologically active metals were likely to be associated with the insoluble fraction. Insoluble heavy metals and dust could also have adverse effects on the human body [41]. In addition, PM_2.5_ could also attach a large number of bacterial microbes. Previous toxicological studies proposed that particulate matter containing more insoluble components from incomplete combustion was more toxic than PM_2.5_ in macrophage and fibroblast cell lines [48,49]. Our experiment showed that, after exposure to PM_2.5_ for 12 h, the levels of IL-6, IL-12 p70, and TNF-α significantly increased in the high dose group of I-PM_2.5_. This indicated that a high dose of I-PM_2.5_ could cause inflammation in a relatively short time. After exposure to I-PM_2.5_, the expression levels of IL-1β, IL-8, IL-12 p70, TNF-α, and CRP increased significantly, and I-PM_2.5_ caused a drastic inflammatory response to macrophages. However, the levels of IL-1β and IL-12 p70 in the mid-dose group did not increase, which may be due to the effect of cell resistance to inflammation or the decrease in cell survival rates. An important inflammatory transmitter, TNF-α plays a crucial role in the initiation and maintenance of inflammation. Increased secretion of TNF-α could induce other pro-inflammatory molecules (such as IL-1, IL-6, and IL-8), amplify inflammatory signals, and cause cascade reactions. When the respiratory system is infected, the expression levels of TNF-α will be significantly increased. Insoluble PM_2.5_ led to high levels of these important inflammatory molecules, such as IL-1β, IL-8, IL-12 p70, and TNF-α, but there was no significant change in IL-6, which was a cause of doubt in this experiment. C-reactive protein has inflammatory marker properties and expresses when acute inflammation occurs. The effect of I-PM_2.5_ on CRP was also consistent with previous studies [50]. A cytokine secreted by macrophages and epithelial cells etc., IL-8 is considered to be a mediator of inflammatory responses [51]. Further, COX-2 is a proinflammatory enzyme that has been reported to be involved in inflammatory responses and cytotoxicity after PM stimulation from different sources [52,53]. Previous research has shown that urban dust or diesel exhaust induce IL-8 and COX-2 in an AhR-dependent manner in human U937-derived macrophages [54,55]. AhR is also considered to be an important receptor protein mediating the toxic responses of dioxin-like PAHs [56]. In our study, exposure to I-PM_2.5_ caused a significant increase in IL-8 and COX-2, and PAHs in I-PM_2.5_ could play a major role. PM_2.5_ caused the occurrence of inflammation and eliminated inflammation through the feedback mechanism. Previous studies have shown that I-PM_2.5_ can cause an increase of ROS at a lower concentration than W- and F-PM_2.5_ [25,57], which indicates that I-PM_2.5_ have a more obvious effect on cytotoxicity and inflammatory reactions. Exposure to PM_2.5_ led to the decrease of IL-2, suggesting that PM_2.5_ might not affect the levels of IL-2 and that changes in IL-2 might be due to the decrease of the cell survival rate.

In our previous study, we found that the overexpression of NF-*κ*B mRNA levels was associated with inflammation and played a role in the development of chronic obstructive pulmonary disease (COPD) in a population experiment [58]. Therefore, we explored whether PM_2.5_ could affect the occurrence and development of respiratory inflammation through such effects in vitro. When cells are in a resting state, the NF-*κ*B signaling pathway is activated by both classical and non-canonical pathways. During the activation of the NF-*κ*B signaling pathway, dimeric protein is transcribed to generate corresponding mRNA. The cytoplasm enters the nucleus rapidly and is specifically bound to the *κ* B site, where the target gene is at the promoter site to regulate its expression [59]. Previous studies have reported that the NF-*κ*B signal pathway could be activated when exposed to PM_2.5_, and it has been significantly correlated with the content of metal elements [34,60]. Moreover, NF-*κ*B plays a dual role in the inflammatory response. Our results show that after exposure to the high dose of W-PM_2.5_, the mRNA expression levels of *RelA*, *RelB*, and c-*Rel* increased. The mRNA levels of the NF-*κ*B signaling pathway genes increased significantly after exposure to F-PM_2.5_. This indicates that the NF-*κ*B signaling pathway responded to the exposure of F-PM_2.5_ at a lower dose, and might lead to subsequent cascade reactions. However, exposure to I-PM_2.5_ had no obvious effect on NF-*κ*B genes, possibly because the excessive accumulation of inflammation destroyed the regulation of the NF-*κ*B signaling pathway.

Studies have shown that the NF-*κ*B family gene is related to the levels of inflammatory molecules—TNF-α, for example, could induce the activation of NF-*κ*B [32]. In addition, the macrophages knockout c-*Rel* and *RelB* lost the function of secreting TNF-α, but oversecreted IL-1β [61]. This indicates that NF-*κ*B can also regulate the expression levels of inflammatory molecules. After exposure to F-PM_2.5_, expression levels of IL-12 p70 showed a positive correlation with NF-*κ*B_2_ and c-*Rel*. After exposure to I-PM_2.5_, IL-1β showed a positive correlation with the mRNA levels of the NF-*κ*B signaling pathway. However, we did not find a correlation between TNF-α and NF-*κ*B, which may be due to the temporality of NF-*κ*B activation.

The source of PM_2.5_ was very wide and its components were complex, which made it difficult to study. The toxic mechanism of macrophages induced by PM_2.5_ is uncertain, because of the interaction between different components. Our subsequent research will focus on the mechanisms of cytotoxicity induced by PM_2.5_.

Although we have done a lot of work, there are still many limitations to our experiment. We only measured the levels of cytokines at 12 and 24 h, and genes at 24 h; thus, some changes in levels could not be fully explained. We only studied changes of mRNA expression levels, and did not observe the expression of NF-*κ*B protein, which is a defect of our study. Because there was no plan to inhibit NF-*κ*B, it is difficult to describe the role of NF-*κ*B genes on the levels of inflammatory molecules. The correlation analysis is different to the causal connection.

## 5. Conclusions

PM_2.5_ decreases the cell survival rate and up-regulates the relative mRNA levels of the NF-*κ*B gene family and the levels of inflammatory molecules. The main toxic components of PM_2.5_ relating to inflammatory response in THP-1-derived macrophages were those of I-PM_2.5_. Exposure to F-PM_2.5_ significantly increased the mRNA expression levels of the NF-*κ*B genes. In addition, the expression levels of inflammatory molecules were correlated with the mRNA expression levels of the NF-*κ*B genes. These findings suggest that PM_2.5_ could up-regulate the relative mRNA levels of the NF-*κ*B family gene and the levels of inflammatory molecules involved in the inflammatory response.

## Figures and Tables

**Table 1:** Concentration of different components of PM_2.5_.

Doses	Concentration of PM_2.5_ (μg/mL)			
	W-PM_2.5_	F-PM_2.5_	I-PM_2.5_	Total PM_2.5_
Low dose	75	25	100	200
Mid dose	150	50	200	400
High dose	300	100	400	800

Note: W-PM_2.5_ = water-soluble PM_2.5_, F-PM_2.5_ = fat-soluble PM_2.5_, I-PM_2.5_ = insoluble PM2.5.

**Table 2:** Primers used in real-time quantitative PCR.

Genes	Primer	Sequence
*β-actin*	Forward	5′-CTGGAACGGTGAAGGTGACA-3′
	Reverse	5′-CGGCCACATTGTGAACTTTG-3′
*NF-κB1*	Forward	5′-CACAAGGCAGCAAATAGACGAG-3′
	Reverse	5′-TGGGGCATTTTGTTGAGAGTT-3′
*NF-κB2*	Forward	5′-GGCTGGTGCTGACATCCAT-3′
	Reverse	5′-CTGCTTCGGGTGTCCTTCTC-3′
*RelA*	Forward	5′-CCCCAGCCCTATCCCTTTAC-3′
	Reverse	5′-TGCCCAGAAGGAAACACCA-3′
*RelB*	Forward	5′-ATGAATGTGGTGAGGATCTGCTT-3′
	Reverse	5′-CTCTGATGTGTTTGTGGATTTCTTG-3′
c-*Rel*	Forward	5′-GACGACTGCTCTTCCTCCTGTT-3′
	Reverse	5′-TCATCTCCTCCTCTGACACTTCC-3′

**Table 3:** Correlation between NF-*κ*B mRNA and inflammatory molecules of macrophage after PM_2.5_ exposure.

Component	Cytokines	IL-1β	IL-6	IL-8	IL-12 p70	CRP	TNF-α
W-PM_2.5_	*NF-κB_1_*	0.211	−0.626	−0.388	0.251	0.055	−0.398
	*NF-κB_2_*	0.460	−0.335	−0.068	−0.221	−0.081	−0.189
	*RelA*	−0.020	−0.330	−0.369	−0.468	−0.289	0.287
	*RelB*	0.500	−0.512	−0.374	−0.259	−0.495	−0.014
	c-*Rel*	0.479	−0.519	−0.278	−0.085	−0.305	−0.030
F-PM_2.5_	*NF-κB_1_*	−0.370	−0.178	−0.584	0.580	0.100	−0.117
	*NF-κB_2_*	−0.440	−0.212	−0.048	0.641 *	−0.372	−0.627
	*RelA*	−0.428	−0.325	−0.164	0.445	−0.466	−0.325
	*RelB*	−0.332	−0.487	−0.129	0.260	−0.203	−0.343
	c-*Rel*	−0.424	−0.263	−0.696 *	0.634 *	0.047	−0.156
I-PM_2.5_	*NF-κB_1_*	0.674 *	−0.695 *	0.605	−0.173	−0.131	−0.424
	*NF-κB_2_*	0.636 *	−0.739 *	0.641 *	−0.256	−0.043	−0.356
	*RelA*	0.681 *	−0.710 *	0.577	−0.167	−0.148	−0.405
	*RelB*	0.322	−0.656 *	0.212	−0.093	−0.175	−0.444
	c-*Rel*	0.700 *	−0.681 *	0.609	−0.149	−0.139	−0.436

* p < 0.05.
